# An Infected Dog Bite With Neisseria animaloris and Neisseria canis Causing Compartment Syndrome, Necrotizing Fasciitis and Gas Gangrene: A Case Report and Review of the Literature

**DOI:** 10.7759/cureus.77296

**Published:** 2025-01-11

**Authors:** James M Comer, Richard Giovane, Luis Pernia

**Affiliations:** 1 Infectious Disease, University of Mississippi Medical Center, Jackson, USA; 2 Family Medicine, University of Alabama, Tuscaloosa, USA; 3 Plastic Surgery, DCH Regional Medical Center, Tuscaloosa, USA

**Keywords:** dog bites, gas gangrene, hand compartment syndrome, necrotizing fascitis, neisseria spp

## Abstract

*Neisseria animaloris* and *Neisseria canis *are rare, zoologic microorganisms that are found in the oral and nasal cavities of felines and canines. Though the pathogenicity of *Neisseria meningitis* and *gonorrhea* are common, *N. canis* and *animaloris* have been far less documented to cause infections in humans and moreover it is exceedingly rare to cause necrotizing fasciitis and gas gangrene. We report the first successful surgical treatment of a 46-year-old female with a case of necrotizing fasciitis, compartment syndrome, and gas formation formed from a bacterial colony involving* Neisseria animaloris, Neisseria canis, Bacillus cereus, Strepococcus viridans, *coagulase-negative *Staphylococcus aureus,* and C*orynebacterium.*

## Introduction

Neisseria is a gram-negative, catalse-negative and oxidase-positive, coccobacillus bacteria that is able to ferment glucose and produce arginine dihydrolase [1,2]. Infection by Neisseria is through the release of peptidoglycan, lipooligosaccharides, and outer membrane vesicles as fragments which induce inflammation and immune response [3]. Although 11 known species of Neisseria exist, humans are mostly susceptible to two strains, N. meningitidis and N. gonorrhea. Neisseria animaloris and Neisseria canis are frequently isolated from the gingiva, oral, and nasal secretion of canines and felines [2,4]. These strains are rare zoonotic pathogens in humans, but they are usually associated with cat or dog bites [1]. Non-gangrenous human wound infections with these bacteria are rare and the literature is sparse with case reports, with only 14 documented cases [5]. No cases have been described with N. animaloris and N. canis causing gangrenous infection with compartment syndrome and necrotizing fasciitis.

Compartment syndrome is a medical emergency and is caused by an increased pressure within a closed muscle compartment which reduces blood flow and can damage muscles and nerves [6]. Patients present with numbness, tingling, pain out of proportion, muscle pain and a decreased pulse. Definitive diagnosis is made by measuring intercompartmental pressure, with >30 mmHg being diagnostic, and treatment is emergency fasciotomy [7]. Gas gangrene is a lethal infection of the skin and soft tissues, with it usually caused by Clostridium species [8]. While the Infectious Disease Society of America refers to gas gangrene as being caused by Clostridium spp., other authors use the term “gas gangrene” to refer to any aerogenic soft tissue infection [9,10].

We report a novel case of a 46-year-old female with gas gangrene, compartment syndrome, and necrotizing fasciitis caused by polymicrobial infection characterized secondary to N. animaloris, N. canis, B. cereus, S. viridans, S. aureus, and Corynebacterium.

## Case presentation

A 46-year-old female with a past medical history of hypertension and diabetes presented to the emergency room after sustaining a dog bite from a pit bull the night prior. The patient reports that she heard dogs barking outside and tried to break up a fight between a neighborhood dog and a pit bull. She did not know the owner of the pit bull. While breaking up the fight, the pit bull bit her left arm. The patient did not seek medical attention at that time due to transportation issues. However, she came to the emergency room the next day due to the area getting more painful and erythematous. The patient was seen and evaluated in the emergency room, and she noted pain in her left forearm initially but then loss of sensation in her entire left forearm soon after arrival.

**Figure 1:**
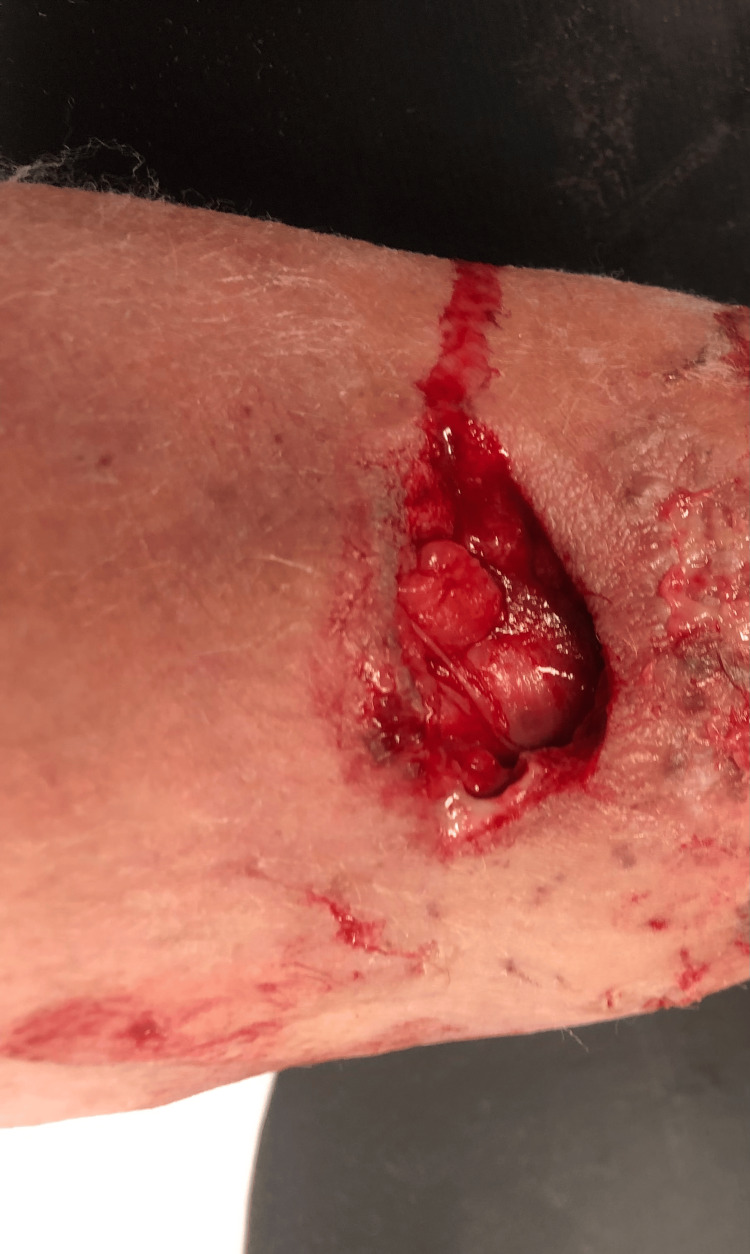
Laceration on left ventral forearm.

On presentation, her vitals were: 98 degrees F, heart rate of 102 BPM, respiratory rate of 20, 155/101 mmHg and breathing at 97% on room air. On physical exam she was noted to have S1S2 with tachycardia, clear breath sounds bilaterally but on her left forearm she was noted to have extensive laceration on left dorsal forearm estimated to be 6 inches in diameter and 2 inches in width with drainage and erythema surrounding borders (Figure 1).

**Figure 2:**
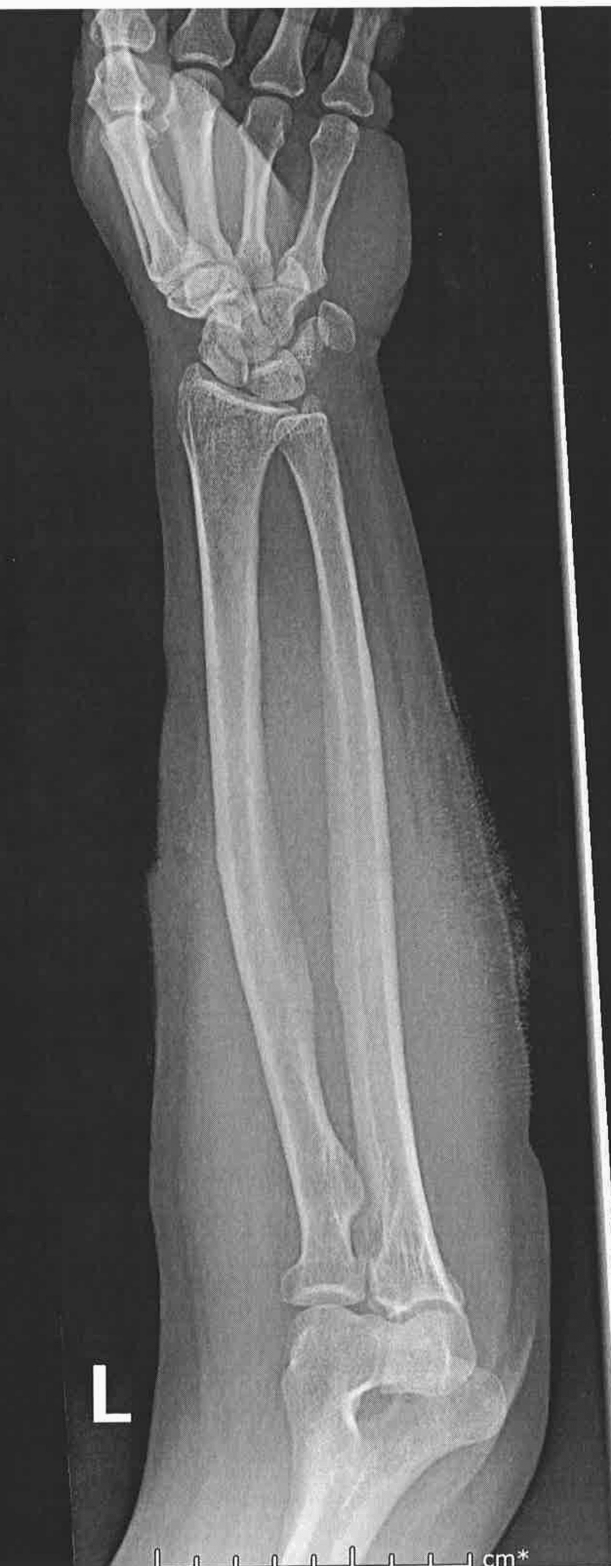
X-ray of left forearm displaying crepitus AP view

**Figure 3:**
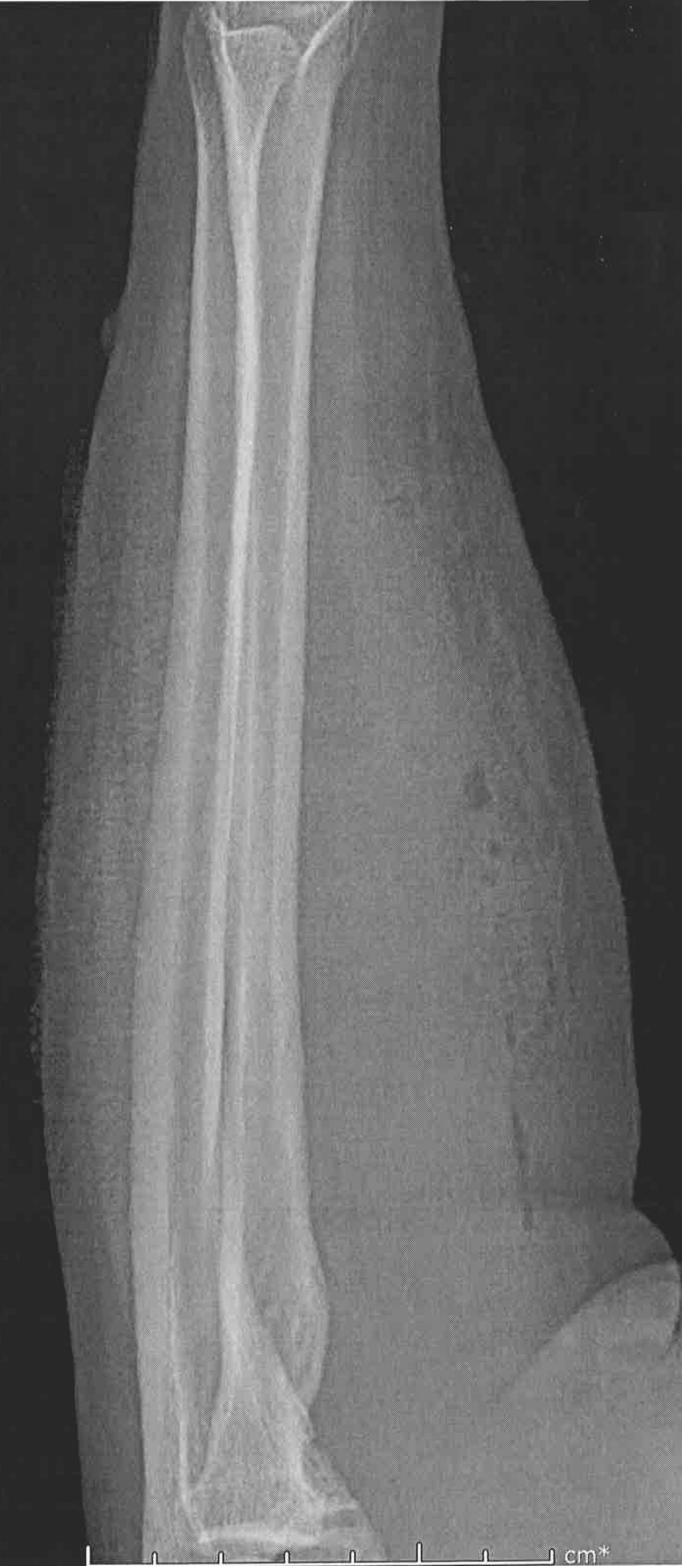
X-ray of left forearm displaying crepitus lateral view

Her distal radial pulses were 2+ bilaterally. The patient had pain with both active and passive flexion and extension of her wrist and elbow. Blood work was done which showed a WBC of 11.44 X10^3/uL. Hemoglobin and hematocrit were within normal limits as were platelets. A comprehensive metabolic panel (CMP) was also done which was only remarkable for a blood glucose of 142 mg/dL. A stat X-ray of the patient’s left forearm was done which showed ventral soft tissue gas scattered throughout the forearm (Figure 2, Figure 3).

There was no radiopaque soft tissue foreign body nor evidence of fracture or other bony abnormality.

The patient was started on 4.5g of piperacillin and tazobactam IV every six hours for broad-spectrum coverage [11]. Plastic surgery was consulted for an emergency wound debridement and fasciotomy due to high suspicion of compartment syndrome. Before the patient went to the operating room, she was counselled extensively on rabies and was offered a rabies vaccine series. The patient declined the series.

**Figure 4:**
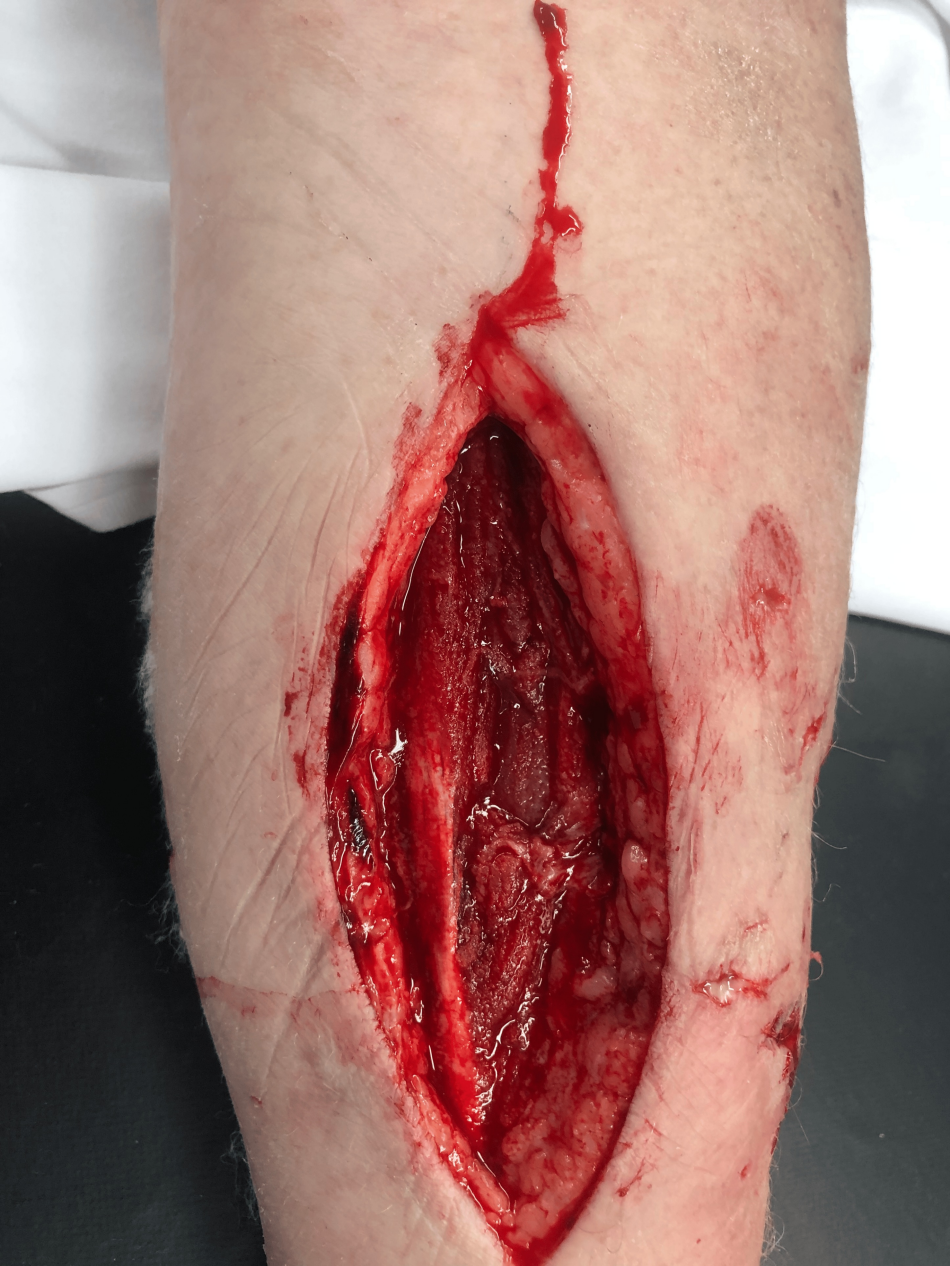
Incision on left ventral forearm

The patient was taken to the operating room for an excisional debridement. It was noted that the bite from the dorsal forearm extended to the volar aspect of her forearm, noting bulging of the muscles against the fascia with discoloration (Figure 4).

A dorsal fasciotomy was done to both extensor compartments. A longitudinal incision was done to the volar forearm noting gas bubble formation with crepitation of the subcutaneous soft tissue and bubbles beneath the forearm fascia. Samples of pus were taken for culture. A black wound vacuum-assisted closure (VAC) dressing of 22cmx14cm was placed on the patient’s left forearm.

Postoperatively, the patient had decreased pain with dorsiflexion of her digits and volar flexion. The patient was continued on IV antibiotics during this time. On hospital day two, the patient was taken back to the OR for further debridement and dressing change. Under loupe magnification, excisional debridement was done, noting that the muscles of the forearm appeared viable and healthy. There was some non-viable fascia that was excised. On hospital day three, cultures of the wound showed growth of N. animaloris, N. canis, B. cereus, S. viridans, S. aureus, and Corynebacterium. At this time, infectious disease was consulted and recommended changing the antibiotics to IV ampicillin/sulbactam with IV clindamycin for broader coverage.

Unfortunately, a MIC was never obtained. The patient, however, remained afebrile during her entire hospitalization, as well as displaying a downward trend in WBC count. The initial WBC was 11.44 X10^3/uL, then 9.8X10^3/uL on day 3 and then ranged from 6.8-7.4 X10^3/uL during the remainder of hospitalization. It should be noted that a complete metabolic panel was done daily and remained unremarkable during hospitalization. The patient continued to work with physical therapy while receiving IV antibiotics. Her pain and mobility improved.

Due to the size of the patient’s wound, she was taken back to the OR on hospital day 8 for skin graft placement using the patient’s left thigh. There were no intraoperative or postoperative complications during this procedure. Postoperatively, the patient was monitored for two more days before being discharged with a seven-day course of amoxicillin/clavulanic acid by mouth (PO) with clindamycin PO. The patient followed up in clinic at one week as well as one month. During these visits the patient reported no pain and had complete functionality of her left forearm.

## Discussion

While several bacterial species are known to cause gas gangrene, clinical settings have reported increasing cases caused by other bacterial species [9]. The case presented is novel because there have been no other reported cases of gas gangrene or necrotizing fasciitis with compartment syndrome due to a polymicrobial infection involving N. animaloris and N. canis. 

Upon review of the literature, a previous case report of an N. animaloris infection shared some characteristics with our case. There was however no evidence of gas gangrene formation [5]. In comparison, neither patient showed growth of Clostridium spp. and both cases used surgical debridement of the wounds for treatment [5]. 

Unfortunately, common traits of Neisseria infections described in the literature are misdiagnosis or a delayed diagnosis, which has led to chronic, non-healing wounds, sepsis, and even death [2,5]. The incidence of N. animaloris remains unknown, however recent literature suggests it may be more common than recently suspected [2].

Gas gangrene is a medical emergency and requires immediate debridement in the operating room with concurrent aggressive IV antibiotics [10,12]. Our case is novel in that the cause of both Neisseria species yielded necrotizing fasciitis, gas gangrene and compartment syndrome which has not been previously described in the literature. In this regard, clinicians should always be suspicious and moreover empirically treat Neisseria when dealing with gas gangrene and necrotizing fasciitis. The success demonstrated in treating an infection with gas formation caused by N. animaloris and N. canis provides further implications in future cases with a similar patient presentation.

## Conclusions

The absence of Clostridium species and the successful management with surgical debridement and aggressive IV antibiotics underscore the importance of prompt and accurate diagnosis in such cases. Clinicians need to maintain a high index of suspicion for Neisseria species when encountering severe infections with gas formation due to the risk of Neisseria causing life-threatening conditions.
